# Genotoxicity and oxidative stress induction by calcium hydroxide, calcium titanate or/and yttrium oxide nanoparticles in mice

**DOI:** 10.1038/s41598-023-46522-0

**Published:** 2023-11-10

**Authors:** Hanan R. H. Mohamed, Ahmed H. Farouk, Salma H. Elbasiouni, Kirolls A. Nasif, Gehan Safwat, Ayman Diab

**Affiliations:** 1 Zoology Department Faculty of Science, Cairo University, Giza, Egypt; 2 Faculty of Biotechnology, October University for Modern Sciences and Arts, October City, Egypt

**Keywords:** Health occupations, Risk factors

## Abstract

Intensive uses of Calcium hydroxide (Ca(OH)_2_NPs), calcium titanate (CaTiO_3_NPs) and yttrium oxide (Y_2_O_3_NPs) nanoparticles increase their environmental release and human exposure separately or together through contaminated air, water and food. However, too limited data are available on their genotoxicity. Therefore, this study explored the effect of Ca(OH)_2_NPs, CaTiO_3_NPs or/and Y_2_O_3_NPs administration on the genotoxicityand oxidative stress induction in mice hepatic tissue. Mice were orally administered Ca(OH)_2_NPs, CaTiO_3_NPs and Y_2_O_3_NPs separately or simultaneously together at a dose level of 50 mg/kg b.w. for two successive weeks (3 days per week). Marked induction of DNA damage noticed after oral administration of Ca(OH)_2_NPs or CaTiO_3_NPs alone together with high Ca(OH)_2_NPs induced reactive oxygen species (ROS) generation and a slight CaTiO_3_NPs induced ROS production were highly decreased after simultaneous coadministration of administration of Y_2_O_3_NPs with Ca(OH)_2_NPs and CaTiO_3_NPs up to the negative control level. Oral administration of Y_2_O_3_NPs alone also did not cause observable changes in the genomic DNA integrity and the ROS generation level compared to the negative control levels. Similarly, significant elevations in P53 gene expression and high reductions in Kras and HSP-70 genes expression were observed only after administration of Ca(OH)_2_NPs alone, while, remarkable increases in the Kras and HSP-70 genes expression and non-significant changes in p53 gene expression were noticed after administration of CaTiO_3_NPs and Y_2_O_3_NPs separately or simultaneously together with Ca(OH)_2_NPs. Conclusion: Ca(OH)_2_NPs exhibited the highest genotoxic effect through oxidative stress induction and disruption of apoptotic (p53 and Kras) and protective (HSP-70) genes expression. Slight DNA damage was noticed after CaTiO_3_NPs administration. However, administration of Y_2_O_3_NPs alone was non-genotoxic and coadministration of Y_2_O_3_NPs with Ca(OH)_2_NPs and CaTiO_3_NPs restored genomic DNA integrity and normal expression of apoptotic p53 and protective HSP-70 genes disrupted by Ca(OH)_2_NPs and CaTiO_3_NPs. Thus co-administration of Y_2_O_3_NPs with Ca(OH)_2_NPs and CaTiO_3_NPs is recommended to counter Ca(OH)_2_NPs and CaTiO_3_NPs induced genotoxicity and oxidative stress.

## Introduction

Nanoparticles have been used to enhance the pharmacokinetic and pharmacodynamics properties of pharmaceutical compounds, and also to improve environmental quality. Nanotechnology research and development has also increased due to its potential, with funding prioritized for societal advancement and sustainable development^1^. For example, calcium hydroxide nanoparticles (Ca(OH)_2_NPs) are used in a variety of applications, such as heritage conservation, deacidifying wood products, preserving cultural heritage structures, and protecting and repairing construction materials^2^. Recently, the potent antimicrobial and penetration activities of Ca(OH)_2_NPs increases its uses in food, medicine and wastewater treatment^3^. Similarly, the high dielectric constant, ferroelectricity, chemical stability, low dielectric loss, low cost, and environmental friend of Calcium titanate (CaTiO_3_NPs) nanoparticle also increases its uses in various biological applications, such as orthopaedic hip replacements and dental implants for increasing the bioactivity of bone-bonding^4^.

Nanomaterials such as yttrium oxide (Y_2_O_3_NPs) have also been used in many applications such as biological imaging, photodynamic treatment, material sciences, and chemical synthesis^5^ because of their high catalytic and penetration activities. Upon on the aforementioned huge uses of Ca(OH)_2_NPs, CaTiO_3_NPs and Y_2_O_3_NPs in food, medicine and other applications, the risk of human exposure to these nanoparticles separately or together in combination raises through various ways including food and contaminated water. However, their genotoxicity have not been well investigated. Two recent studies by Mohamed^6,7^ manifested the induction of DNA breaks in the liver, brain, lung and other organs of mice given a single oral dose (100 mg/kg) of Ca(OH)_2_NPs through the excessive generation of ROS, mitochondrial DNA damage and alterations of the expression of the apoptotic genes and inflammatory cytokines. The cytotoxicity of CaTiO_3_NPs has been demonstrated in vitro on normal human skin fibroblast cells^8^, but almost no in vivo studies have been conducted.

As a result of above mentioned huge uses of Ca(OH)_2_NPs, CaTiO_3_NPs and Y_2_O_3_NPs their environmental release and human exposure separately or together increase through contaminated air, water and food, along with the responsibility of liver for immunity, digestion, detoxification, metabolism, and vitamin storage increases risk of nanoparticles and other chemicals on liver integrity and function^2,8,9^. However, the in vivo genotoxicity of Ca(OH)_2_NPs, CaTiO_3_NPs or/and Y_2_O_3_NPs have been poorly studied. Consequently, the present study was conducted to estimate the effect of Ca(OH)_2_NPs, CaTiO_3_NPs or/and Y_2_O_3_NPs administration on the genomic stability and ROS generation in mice liver tissue. Genomic stability was assessed using the alkaline Comet assay, and the level of ROS generation within hepatocytes was studied using 2,7- dichlorofluorescein diacetate (DCFH-DA)**.** Quantitative real-time polymerase chain reaction (qRTPCR) was also done to measure expression of some genes.

## Materials and methods

### Chemicals

The three tested nanoparticles were obtained in the form of nano-powders with size less than 100 nm. The nano-powders of CaTiO_3_NPs and Y_2_O_3_NPs were purchased from Sigma Aldrich Company (St. Louis, MO, USA) with about 99% trace metals and product code number of 633801 and 544892, respectively, while, Ca(OH)_2_NPs were purchased from Nanotech Company (Giza, Egypt). The size, shape and dispersion of Ca(OH)_2_NPs, CaTiO_3_NPs and Y_2_O_3_NPs have been well characterized l in our previous studies^5,8,10^.

### Animals

The MSA University Research Ethics Committee authorized the study's design including the use of 45 male Swiss webster mice, each weighing 20–25 g and aged 10 to 12 weeks. The mice were obtained from the National Research Center (Giza, Egypt) and housed in the Zoology department's animal house at Cairo University for three weeks under regular circumstances. The experiment lasted 4 weeks, during which time they were provided conventional food pellets and water.

### Ethical approval

The experimental design of the presented study was documented by the MSA University Research Ethics Committee. This study was conducted according to ARRIVE guidelines and also Animal handling and experimentations were performed in according to the Guidelines of the National Institutes of Health (NIH) regarding the care and use of animals for experimental procedures.

### Determination of the nanoparticles tested dose

Acute toxicity test was done to determine the proper dosage of the tested three nanoparticles according to OECD Standard 420 guidelines. Four groups of five mice each; negative control group and three test groups were created. The three test groups were orally received a suspension containing 2000 mg/kg of Ca(OH)_2_NPs, CaTiO_3_NPs or Y_2_O_3_NPs once a time, while, negative control group received the exact same amount of deionized distilled water. Mice of all groups were carefully noticed for signs of toxicity and death during the first 24 h and until 14 days after administration. Considering the mice's survivability and OECD guidelines 420^11,12^, the tested dose of Ca(OH)_2_NPs, CaTiO_3_NPs and Y_2_O_3_NPs was considered as 2½ of the determined safe dose.

### Experimental design

Twenty five male mice were randomly divided into five groups of five mice each. The first group served as the negative control and orally given deionized distilled water, while, the remaining four groups orally received a suspension of Ca(OH)_2_NPs, CaTiO_3_NPs or Y_2_O_3_NPs at a dose level of 50 mg/kg separately or simultaneously together three times each week for a period of two weeks. The mice of five groups were then killed by neck dislocation, and their liver tissues were removed, stored, and frozen at − 80 °C for future research.

### Alkaline comet assay

Using an alkaline comet assay with a pH level greater than 13, DNA damage was determined in the negative control and treated groups according to protocol described by Tice et al.,^13^. Slides were photographed using the epi-fluorescent microscope (OLYMPUS CKX 41) at 200× magnification, then the photographed comet nuclei were analyzed and scored using the TriTek Comet ScoreTM Freeware v1.5.

### Genes expression

Quantitative real time polymerase chain reaction (qRTPCR) was done to measure the mRNA expression level of p53, Kras and HSP-70 genes in liver tissues of the studied five groups using the pre-designed primers sequences listed in Table 1^14–16^. The mRNA expression level of the housekeeping β-actin gene was used to calibrate the expression of the three studied genes, and the comparative Ct method was used to determine fold change in gene expression.

**Table 1:** Sequences of the primers used in qRT-PCR.

Gene	Strand	Sequence
P53	Forward	5′-ACCATCGGAGCAGCCCTCAT-3′
	Reverse	5′-TACTCTCCTCCCCTCAATAAG-3′
Kras	Forward	5-TATAAACTTGTGGTGGTTGGAGCT-3
	Reverse	5-GTACTCATCCACAAAGTGATTCTG-3
*hsp70*	Forward	5′-TGGTGCTGACGAAGATGAAG-3′
	Reverse	5′-AGGTCGAAGATGAGCACGTT-3′
β-actin	Forward	5′-TCACCCACACTG TGCCCATCT ACG A-3′
	Reverse	5′-GGATGCCACAGGATTCCATACCCA-3′

### Generation of reactive oxygen species (ROS)

The level of reactive oxygen species (ROS) generation within liver tissues was studied using the highly specific 2,7dichlorofluorescein diacetate (DCFH-DA) dye for ROS^17^. A 50 µl of the cell suspension was mixed with 50 µl of DCFH-DA (20 mM), the mixture was incubated in the dark for 30 min the mixture was then placed on clean slides and finally imaged under an epi-fluorescent microscope (OLYMPUS CKX 41) at 200× magnification.

### Statistical analysis

Results of Comet assay and RTPCR were displayed as mean ± Standard Deviation (SD) and were evaluated using SPSS (version 20) at a significance level of < 0.05. One-way analysis of variance (ANOVA) was used to determine the impact of Ca(OH)_2_NPs, CaTiO_3_NPs or/and Y_2_O_3_NPs administration on DNA damage induction and expression level of p53, Kras and HSP-70 genes. Duncan's test was carried out to determine the similarities and differences between the control and four treated groups.

## Results

### Characterization of Ca(OH)_2_NPs, CaTiO_3_NPs and Y_2_O_3_NPs

Characterization of Ca(OH)_2_NPs, CaTiO_3_NPs and Y_2_O_3_NPs in our previous studies using XRD analysis, particle size distribution and Transmission electron microscope (TEM) confirmed the purity of purchased nanopowders along with stability and well distribution of suspended nanoparticles in deionized distilled water. Moreover, TEM imaging revealed the spherical morphology of Ca(OH)_2_NPs, CaTiO_3_NPs and Y_2_O_3_NPs^5,8,10^.

### Nanoparticles’ tested dose

Observing the mice after oral intake of Ca(OH)_2_NPs, CaTiO_3_NPs or Y_2_O_3_NPs separately at a dose level 2000 mg/kg revealed that all mice were still healthy and no signs of toxicity were seen during the frst 48 h of nanoparticles administration until the end of the 14-day observation period. The half lethality dose (LD50) of Ca(OH)_2_NPs, CaTiO_3_NPs and Y_2_O_3_NPs was thus considered to be above 2000 mg/kg according to the OECD-420 guidelines, and the tested dose of nanoparticles in this study was calculated as 2½ equals to 50 mg/kg body weight of the LD50 obtained from acute toxicity test.

### Comet assay

**Figure 1:**
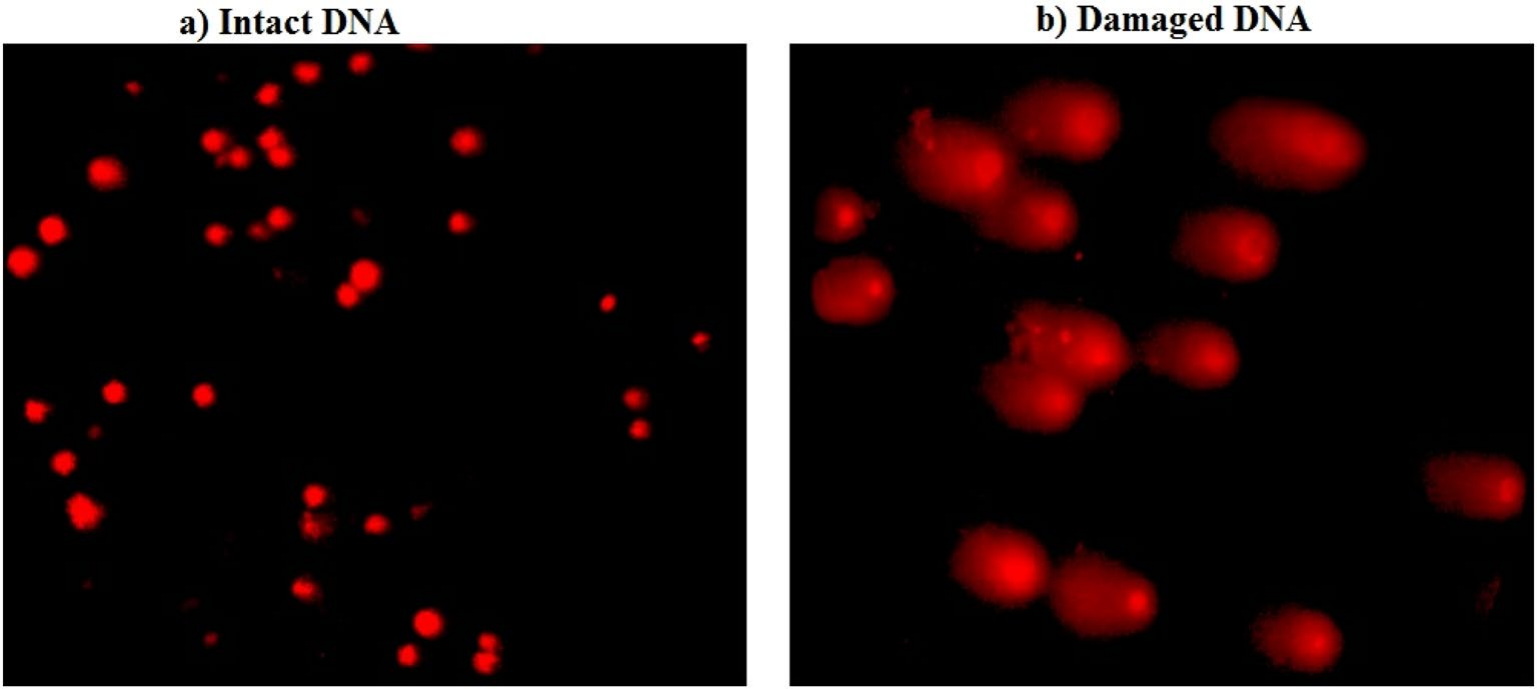
Representative examples for scored Comet nuclei with (**a**) intact and (**b**) damaged nuclei regardless of treatment.

The results of the Comet assay are summarized in Table 2 and showed that oral administration of Ca(OH)_2_NPs three times a week for two weeks at the dose level of 50 mg/kg b.w (Group II) caused statistically significant elevations in the tail length, %DNA in tail and tail moment compared to the negative control (Group I) and the other three treated (Groups III, IV & V) groups. On the other hand, tail length and tail moment were non-statistically significantly altered and remained in the negative control level in the liver tissues of mice orally given CaTiO_3_NPs (Group III), or Y_2_O_3_NPs (Group IV) separately or simultaneously with Ca(OH)_2_NPs (Group V) as displayed in Table 2. However, oral administration of CaTiO_3_NPs (Group III) alone or in combination with Ca(OH)_2_NPs and Y_2_O_3_NPs (Group V) caused marked elevations in the %DNA in tail compared to the negative control values (Table 2). Examples of examined and scored Comet nuclei with intact and damaged DNA are displayed in Fig. 1.

**Table 2:** Tail length (px), %DNA in tail and tail moment in the liver tissue of the negative control group and groups administered Ca(OH)_2_NPs, CaTiO_3_NPs and Y_2_O_3_NPs.

Group	Treatment	Tail length (px)	%DNA in tail	Tail moment
I	Negative control	6.13 ± 1.38^a^	27.44 ± 8.41^a^	2.08 ± 1.07^a^
II	Ca(OH)_2_NPs	14.93 ± 0.92^b^	30.01 ± 3.35^ab^	4.59 ± 0.63^b^
III	CaTiO_3_NPs	7.21 ± 0.06^a^	37.94 ± 3.87^b^	3.39 ± 0.30^ab^
IV	Y_2_O_3_NPs	6.05 ± 1.68^a^	23.29 ± 4.44^a^	1.73 ± 0.88^a^
V	Combination	6.42 ± 0.95^a^	28.73 ± 2.99^ab^	2.04 ± 0.44^a^

Results are expressed as mean ± SD.

Results were analyzed using one-way analysis of variance followed by Duncan’s test to test the similarity between the control and three treated groups.

Means with different letters indicates statistical significant difference between the compared groups in the same column.

### Gene expression

Interpretation of the qRTPCR results revealed that oral administration of Ca(OH)_2_NPs at a dose of 50 mg/kg b.w (Group II) resulted in statistically significant decreases in the expression level of Kras and HSP-70 genes and a marked elevation in the p53 gene expression level compared to their expression level in the liver tissue of negative control (Group I) group and groups given orally CaTiO_3_NPs (Group III), or Y_2_O_3_NPs (Group IV) separately or together simultaneously with Ca(OH)_2_NPs (Group V) as shown in Table 3. On the contrary, the expression level of HSP-70 gene was significantly upregulated after multiple administration of CaTiO_3_NPs (Group III), or Y_2_O_3_NPs (Group IV) separately or together simultaneously with Ca(OH)_2_NPs (Group V) compared to the expression level of Ca(OH)_2_NPs (Group II) and even was significantly higher than the expression level of the negative control (Table 3).

**Table 3:** Expression level of Kras, HSP-70 and p53 genes in the liver tissue of the negative control group and groups administered Calcium hydroxide, calcium titanate and yttrium oxide nanoparticles.

Group	Treatment	Kras	HSP-70	P53
I	Negative control	1.00 ± 0.00^a^	1.00 ± 0.00^a^	1.00 ± 0.00^a^
II	Ca(OH)_2_NPs	0.059 ± 0.03^b^	0.69 ± 0.06^b^	3.07 ± 0.12^b^
III	CaTiO_3_NPs	1.11 ± 0.02^c^	1.64 ± 0.10^c^	1.16 ± 0.15^a^
IV	Y_2_O_3_NPs	1.21 ± 0.02^d^	1.92 ± 0.34^dc^	0.99 ± 0.12^a^
V	Combination	0.90 ± 0.02^e^	1.54 ± 0.10^c^	1.10 ± 0.18^a^

Results are expressed as mean ± SD.

Results were analyzed using one-way analysis of variance followed by Duncan’s test to test the similarity between the control and three treated groups.

Means with different letters indicates statistical significant difference between the compared groups in the same column.

Likewise, the expression level of the Kras gene was markedly elevated after multiple oral administration of CaTiO_3_NPs (Group III), or Y_2_O_3_NPs (Group IV) separately or together simultaneously with Ca(OH)_2_NPs (Group V) compared with the expression level of Ca(OH)_2_NPs (Group II), but still significantly lower than the expression level of negative control group in liver tissue of mice given Ca(OH)_2_NPs**,** CaTiO_3_NPs and Y_2_O_3_NPs (Group V) together simultaneously (Table 3). Meanwhile, the expression level of p53 gene was highly downregulated after multiple oral administration of CaTiO_3_NPs (Group III), or Y_2_O_3_NPs (Group IV) separately or together simultaneously with Ca(OH)_2_NPs (Group V) compared to the expression level of Ca(OH)_2_NPs (Group II) group and even reached the negative control expression level (Table 3).

### Generation of hepatic ROS

**Figure 2:**
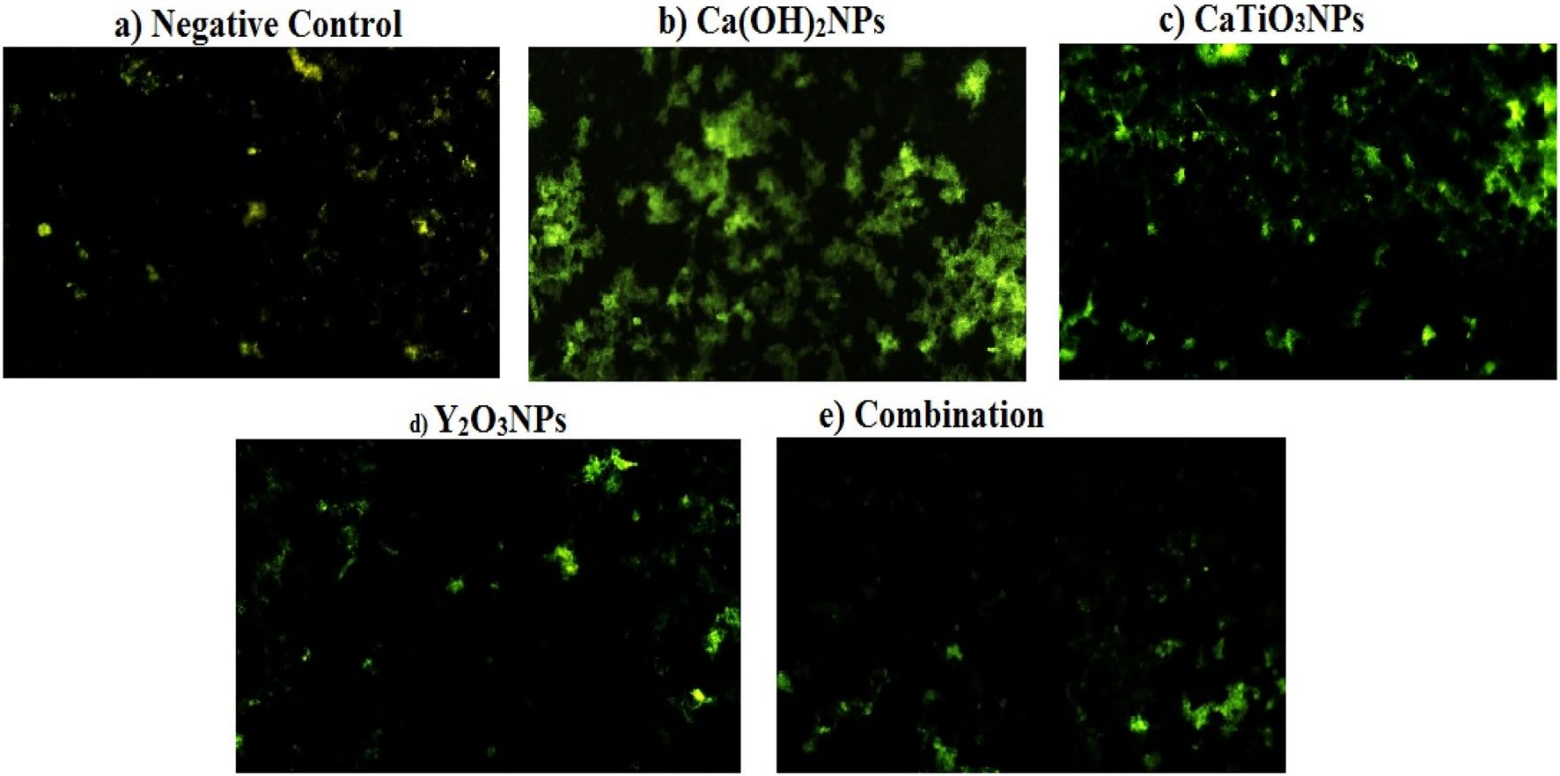
Level of ROS generation within liver cells of (**a**) negative control group and groups given (**b**) Ca(OH)_2_NPs, or (**c**) CaTiO_3_NPs or (**d**) Y_2_O_3_NPs separately or (**e**) together simultaneously.

As shown in Fig. 2 multiple oral administration of Ca(OH)_2_NPs (Group II) for two weeks at the 50 mg/kg dose level led to the highest elevation in hepatic ROS generation compared to their generation within hepatocytes of the negative control group (Group I) and the groups given CaTiO_3_NPs (Group III) Y_2_O_3_NPs (Group IV) separately or together simultaneously (Group V). On the contrary, non-observable changes were noticed in the level of ROS generated within the hepatocytes of mice given orally Y_2_O_3_NPs alone (Group IV) or in combination with Ca(OH)_2_NPs and CaTiO_3_NPs (Group V) compared to the negative control ROS level (Fig. 2). Meanwhile, slight elevation in the ROS level was noticed after multiple administration of CaTiO_3_NPs (Group V) compared with the ROS generation level of the negative control (Fig. 2).

## Discussion

Recent days have witnessed a rapid development of nanotechnology and the use of many nanoparticles in many fields, which increases human exposure and risks. For example, Ca(OH)_2_NPs, CaTiO_3_NPs and Y_2_O_3_NPs recently received great attention for their distinctive properties and have been used in many industries, food and medicine, which increases human exposure through contaminated food and water. However, the effect of Ca(OH)_2_NPs, CaTiO_3_NPs or/and Y_2_O_3_NPs on genomic DNA integrity and oxidative stress induction has not been well studied. Therefore, this study was undertaken to estimate the influence of Ca(OH)_2_NPs, CaTiO_3_NPs or/and Y_2_O_3_NPs administration on the integrity of genomic DNA and level of ROS generation in mice liver tissues.

The alkaline Comet assay detects single- and double stranded DNA breaks with high sensitivity in individual cells^13^. Thus, our detection of remarkable high elevations in the Comet parameters: tail length, %DNA in tail and tail length after Ca(OH)_2_NPs administration confirmed the induction of DNA breakages in the liver tissues of mice given Ca(OH)_2_NPs. These findings supported the reported induction of DNA breakage by a single oral administration of Ca(OH)_2_NPs in the liver, kidney, brain, lung, heart and spleen tissues of mice^5,7^.

On the other hand, administration of CaTiO_3_NPs or Y_2_O_3_NPs separately or simultaneously together with Ca(OH)_2_NPs was safe and non-genotoxic as manifested by the non-remarkable changes observed in the DNA damage parameters measured by Comet assay compared to the negative control values. Although too limited data are available on the CaTiO_3_NPs and Y_2_O_3_NPs genotoxicity, an in vitro recent study of Mohamed et al.,^8^ similarly demonstrated that CaTiO_3_NPs is safe and non-genotoxic to normal human skin fibroblast (HSF) cells.

Generation of ROS occurs normally within cells and acts as messengers for many physiological processes. However, ROS are overproduced upon various stimuli such as the induction of DNA breaks thereby high DNA breakage causes ROS over-generation^5,7,17^. Consequently, the apparent extra production of ROS in this study after Ca(OH)_2_NPs administration within liver cells revealed by high emission of fluorescent light can be attributed to the aforementioned induction of DNA breakage. On the contrary, the non-remarkable changes demonstrated in the level of ROS generated within liver cells of mice given orally CaTiO_3_NPs or Y_2_O_3_NPs separately or in combination with Ca(OH)_2_NPs through the non-observable changes in the intensity of emitted fluorescent light confirmed the above mentioned preservation of genomic DNA integrity consistently with previous studies^8^.

Elevated ROS generation and DNA breakages also induce apoptosis^18^. DNA breakage in particular double stranded-DNA breaks are serious and fatal DNA damage since a one double stranded-DNA break is sufficient to disturb genomic integrity or kill cell^19^ as well as these DNA breaks activate the tumor suppressor p53 gene mediating apoptosis^20,21^. The p53 tumor suppressor gene is activated by various stimuli e.g. DNA breaks, oxidative stress, and activate oncogenes producing p53 protein that triggers apoptosis^22,23^. Additionally, p53 protein is a downstream regulatory factor of the Kras oncogene, a member of the RAS family, inducing apoptosis because overexpression of p53 gene and accumulation of p53 protein that downstream the expression of the anti-apoptotic Kras gene^24,25^. Accordingly, the above mentioned Ca(OH)_2_NPs induced over ROS generation and DNA breaks trigger apoptosis of hepatic cells through marked upregulation of the apoptotic p53 gene and downregulation of anti-apoptotic Kras gene noticed after administration of Ca(OH)_2_NPs.

In contrast, oral administration of CaTiO_3_NPs or Y_2_O_3_NPs separately or in combination with Ca(OH)_2_NPs caused non-s significant changes in the expression level of the p53 gene and significant elevations in the expression level of the Kras gene that motivates hepatic cells repair and inhibits apoptosis because Kras gene overexpression elevates the intracellular RAS protein that down regulates apoptosis and maintains cell survival^23,26^.

HSPs are constituently produced within cells acting as cell protective proteins, therefore, expression of HSPs genes increases dramatically when the cells exposed to different stress e.g. oxidative stress, heat shock and accumulation of various chemicals^27^. In contrast, a marked decrease in the expression level of HSP-70 gene was observed after Ca(OH)_2_NPs administration. This decrease could be explained by excessive ROS generations induced by Ca(OH)_2_NPs because highly generated ROS attack and damage cellular lipids, proteins, carbohydrate and DNA altering their functions and disrupt the equilibrium between oxidants and antioxidants inducing oxidative stress^27,28^.

Ongoing with previous results, multiple administration of CaTiO_3_NPs or Y_2_O_3_NPs separately or simultaneously together with Ca(OH)_2_NPs highly increases the expression level of HSP-70 gene that protects hepatic cells and maintains cells vaibility because overexpression of HSP-70 gene highly elevates HSP-70 that inhibits both intrinsic and extrinsic apoptotic pathways provides cell survival allowing cells repair^29–31^. Moreover, the recently reported antioxidative and free radical scavenging activities of Y_2_O_3_NPs may explain the enhanced antioxidant status of hepatic cell and declined Ca(OH)_2_NPs induced DNA damage observed in mice given Y_2_O_3_NPs with CaTiO_3_NPs or Ca(OH)_2_NPs^6,32^.

## Conclusion

Based on the above findings, Ca(OH)_2_NPs is highly genotoxic and caused genomic DNA damage through over ROS generation and apoptosis induction. However, administration of CaTiO_3_NPs and Y_2_O_3_NPs with Ca(OH)_2_NPs motivated antioxidant status and diminished Ca(OH)_2_NPs induced DNA damage. Moreover, oral administration of CaTiO_3_NPs caused slight DNA damage and Y_2_O_3_NPs was safe and non-genotoxic. Therefore, more studies are recommended to study the Ca(OH)_2_NPs induced genotoxicity and the possibility of using CaTiO_3_NPs and Y_2_O_3_NPs to diminsh Ca(OH)_2_NPs induced negative health effects for human.

## Data Availability

The datasets used and/or analyzed during the current study are available from the corresponding author on reasonable request.
